# Coexistence of Antiphospholipid Syndrome and Heparin-Induced Thrombocytopenia in a Patient with Recurrent Venous Thromboembolism

**DOI:** 10.1155/2017/3423548

**Published:** 2017-04-26

**Authors:** Samuel Adediran, Nicole Agostino

**Affiliations:** Lehigh Valley Health Network, Departments of Hematology and Oncology and Medicine, Allentown, PA, USA

## Abstract

Heparin-induced thrombocytopenia (HIT) is a prothrombotic adverse drug reaction in which heparin forms complexes with platelet factor 4 forming neoantigens that are recognized by autoantibodies. Antiphospholipid syndrome (APS) is similar to HIT in that it is mediated by autoantibodies that are also prothrombotic. We present a case of rare coexistence of antiphospholipid antibody syndrome and heparin-induced thrombocytopenia.

## 1. Introduction

Heparin-induced thrombocytopenia (HIT) is a prothrombotic adverse drug reaction in which heparin forms complexes with platelet factor 4 forming neoantigens that are recognized by autoantibodies (antiHPF4/HAPA). The Fc portions of the anti-HPF4 bind Fc receptors on platelets causing platelets activation, aggregation, release of *α* and dense granules, and formation of procoagulants [1]. The hallmark of this is thrombocytopenia and thrombosis. HIT occurs in about 2% of all patients who receive heparin of whom about 35% develop thrombosis [2]. Antiphospholipid syndrome (APS) is similar to HIT in that it is mediated by autoantibodies that are also prothrombotic. Autoantibodies are generated to phospholipids or to phospholipid-binding proteins which are recognized risk-factors for thrombosis and pregnancy morbidity. Diagnosis of APS requires the elevation of at least one of the phospholipid autoantibodies and a clinical manifestation (Procedure 1). In this report we present a patient with recurrent venous thromboembolism despite being on full anticoagulation who was found to have concurrent HIT and APS.

## 2. Case Report

A 37-year-old Caucasian female with history of obesity and iron-deficiency anemia presented with painful left lower extremity swelling. A physical examination revealed an edematous, tender, and mildly erythematous left lower extremity. Mild tachycardia was noted. A lower extremity venous duplex ultrasound showed an extensive, occlusive deep venous thrombosis (DVT) from the left common femoral vein to the calf veins. Also, there was occlusive DVT within the left common and external iliac veins. A computer tomographic (CT) imaging of the chest with contrast revealed pulmonary emboli within the subsegmental pulmonary artery branches in the right lower lobe. Heparin was administered intravenously. Catheter-directed venous thrombolysis was done, and complete clearing of the left iliofemoral DVT was achieved. Intravenous heparin was switched to rivaroxaban, and the patient was discharged home. A week later, the patient presented with right-sided pleuritic chest pain. A CT of the chest with contrast revealed new acute pulmonary emboli within the distal left lower lobar artery and the basilar segmental pulmonary arteries of the left lower lobe. A venous duplex ultrasound showed extensive left lower extremity DVT from the left common femoral vein to the calf veins and occlusive thrombus within the left external iliac vein and the left common iliac vein. Intravenous heparin drip was initiated. Inferior vena cava filter was placed. Catheter-directed venous thrombolysis was performed, and complete thrombolysis of the left lower extremity DVT was noted within 24 hours. Three days later, heparin was stopped, and a low molecular weight heparin, lovenox, was started. Within 24 hours of starting lovenox, the patient noticed increased left leg tightness and worsening pain. A venous duplex ultrasound was performed and again revealed extensive, occlusive DVT from the left common femoral vein to the left popliteal vein and a nonocclusive DVT in a small left external iliac vein. By this time, platelet count had decreased from 448 × 109/L at the time of admission to 147 × 109/L (normal, 150–400 × 109/L). Enzyme-linked immunosorbent assays (ELISA) were performed in order to evaluate for the presence of heparin associated platelet antibodies (HAPA) and antiphospholipid antibodies (APLA). Lovenox was stopped, and argatroban was started. TPA thrombolysis with aspiration thrombectomy was performed, and a follow-up venography demonstrated complete removal of thrombus from the popliteal, femoral, and common femoral veins. Three days later, while the patient was on argatroban, a venous duplex ultrasound again demonstrated extensive, occlusive DVT in the distal veins (posterior tibial, peroneal, and gastrocnemius) extending to the popliteal, femoral, deep femoral, and common femoral veins. Argatroban was stopped; arixtra, 10 mg daily, was started, and monitoring with serial venous duplex ultrasound was continued. Three days after starting arixtra, partial recanalization of DVT within the left external iliac vein and the left femoral popliteal system was demonstrated. Platelet count nadir at 134 × 109/L. HAPA was strongly positive 1.412 (normal, <0.4 OD). Serotonin release assay was positive with 82% (normal, ≥20%) serotonin release with low-dose heparin (0.1 U/mL) and 0% (normal, =20%) serotonin release with high-dose heparin (100 U/mL). Antiphospholipid antibody profile demonstrated elevated anti-*β*2 glycoprotein 1, anticardiolipin, and positive lupus anticoagulant (Table 1). Once platelet count stabilized and DVT recanalization was achieved, Coumadin was started. Coumadin was overlapped with arixtra for 5 days after INR reached therapeutic level. Three months after the initial hospital encounter, antiphospholipid antibodies levels were still elevated, confirming the diagnosis of antiphospholipid syndrome. We recommended against pregnancy giving her significant thrombotic risk, and the risk of fetal toxicity with Coumadin.

## 3. Discussion

The coexistence of antiphospholipid antibody syndrome and clinical heparin-induced thrombocytopenia is not often seen. This case demonstrates the simultaneous occurrence of both entities in an otherwise healthy patient. A similar case was reported by Tun et al. wherein a 42-year-old female found to have pulmonary embolism, superior mesenteric artery thrombus, and multiple infarctions had thrombocytopenia and positive HAPA. However, serotonin release assay was not reported [3]. While HAPA as measured by ELISA is very sensitive, serotonin release assay, a functional assay that determines the ability of heparin-dependent IgG to activate platelet to secrete serotonin, is more specific. Correlation between APS and HAPA/anti-HPF4 has been suggested. Lasne et al. reported twenty patients with APS three of whom tested positive for HAPA. One of the patients had positive serotonin release 26% (normal, ≥20%). None of these patients reported history of HIT or received heparin in at least thirty months. They suggested that APS may increase predisposition to HIT [4]. In a study in which sixty-nine patients had positive APLA with or without APS, ten patients (14.5%) were reported positive for HAPA, three of whom had prior heparin exposure. No HIT was reported [5]. Another small retrospective study reported that out of thirty-three patients with APLA, twenty of whom had primary APS, seven developed HAPA. Two of the seven patients had positive aggregation tests concerning for HIT. Two had no prior exposure to heparin [6]. It has been proposed that the distortion of vascular endothelium seen in APS causes the exposure of glycosaminoglycans such as heparin sulfate that may then complex with platelet factor 4. This may cause molecular modification that produces neoantigens and HAPA [7].

On the other hand, Martin-Toutain et al. found poor correlation between two different ELISA tests for HAPA on the same blood sample. This prompted them to suggest that the presence of HAPA may be related to cross-reactivity between APLA and antibodies used in various ELISA and not necessarily a pathogenic antibody responsible for development of clinical HIT [8]. Martinuzzo et al. found no cross-reactivity between HAPA and IgG anticardiolipin or IgG anti-*β*2-glycoprotein 1 [6]. Other studies have shown partial cross-reactivity [9]. Therefore, it is still not clear whether or not the presence of APLA predisposes a patient to developing HAPA. To further complicate matters, there are at least two reported cases of heparin-related thrombocytopenia in patient with APS who developed thrombosis but had negative HAPA. It was suggested that the presence of antiphospholipid antibodies possibly interferes with HIT diagnostic tests causing a lack of antigen recognition by the antibodies used in the tests [10, 11]. Our patient clearly had elevated antiphospholipid antibodies, markedly positive HAPA, and high positive serotonin release. Thus, it represents a true case of concurrent antiphospholipid syndrome and clinical heparin-induced thrombocytopenia in an otherwise healthy patient.

The mainstay of treatment for venous thromboembolism (VTE) secondary to APS is heparin in the acute setting and vitamin K antagonist (VKA) in the long term. Warfarin has numerous dietary and drug interactions and there is need for frequent monitoring of INR making its use somewhat challenging. Also, variable responsiveness of reagents used in INR test to lupus anticoagulant can complicate monitoring. As a result, the use of new oral anticoagulants (NOACS) like dabigatran, apixaban, and rivaroxaban has been advocated. However, there are no good data to recommend the use of NOACs in APS [12]. A few case reports have shown VTE recurrences in patients with APS who were treated with rivaroxaban and dabigatran, indicating that NOACS may not be as effective as VKAs in APS [13]. After our patient's first episode of pulmonary embolism and DVT, she was discharged home on rivaroxaban. She presented a week after with extension of DVT and PE. In retrospect, this was likely a failure of rivaroxaban in APS. Positive serotonin release confirmed HIT, indicating that two process were probably happening concurrently. Despite no good supporting data, some have suggested that it is reasonable to consider NOACs in patients who do not tolerate VKAs [12]. In a retrospective review of thirty-five patients with APS who were switched to rivaroxaban after VKA intolerance, no recurrent VTE was observed after a median follow-up of ten months [14]. The ongoing prospective randomized control trial “rivaroxaban in antiphospholipid syndrome” will shed more light on the use of rivaroxaban as compared to VKAs in the secondary prevention of VTE in antiphospholipid syndrome.

Heparin-induced thrombocytopenia is treated by stopping heparin and administering non-heparin anticoagulants. Argatroban is the only FDA-approved anticoagulant for this indication. However, other non-heparin anticoagulants, namely, bivalirudin, fondaparinux, and danaparoid, have been used successfully. Warkentin et al. reviewed sixteen patients with HIT who were treated with fondaparinux. None of the patients developed new, recurrent, or progressive thrombosis. A few other reports have described successful treatment of HIT with fondaparinux [15].

In a patient with both HIT and APS, the 14th international congress on APL task force suggests anticoagulation with argatroban or danaparoid. The use of danaparoid has been discontinued in the United States. Our patient received argatroban, but her clot burden worsened necessitating a change to fondaparinux. We found four other case reports in the literature, detailing the successful use of fondaparinux in patients with concurrent APS and HIT [16–18]. Two of these cases were pediatric. Our case further adds to the point that fondaparinux can be successfully used to treat VTE in patient with HIT and APS at least in the acute setting.

For a child-bearing female with history of HIT and APS who requires indefinite anticoagulation, the choice of anticoagulant to use during pregnancy is complicated. The only anticoagulant recommended during pregnancy for a patient with APS is low molecular weight heparin (LMWH). However, LMWH is better avoided in HIT. Warfarin is contraindicated in pregnancy due to high risk of fetal malformations especially in the first trimester [19]. No good data exist on the potential risks of the NOACs in pregnancy. Animal studies have shown reproductive toxicity, embryo-fetal toxicity, and increase incidence of common malformations with the NOACs. Because the potential risk of NOAC in human is unknown, they should be avoided during pregnancy and breastfeeding [20]. Other treatment considerations in pregnancy are fondaparinux and danaparoid. There is no good quality data to support their use in pregnancy. However there are a few case reports and case series about the safety of fondaparinux in pregnancy. A retrospective study reviewed 13 women with 15 pregnancies who were treated with fondaparinux because of hypersensitivity to LMWH. For six of the pregnancies, fondaparinux was started in the first trimester, in the second trimester for eight, and in the third trimester for one. Five pregnancy complications were observed: two miscarriages, one voluntary termination due to congenital malformation, one cerebral palsy due to delay in delivery, and one recurrent DVT due to drug underdosing [21]. A prospective study of twelve pregnancies showed no adverse effect from fondaparinux [22]. It is also worth noting that while Coumadin has been successfully used in some pregnant women with mechanical heart valves, its use is associated with significant complications such has embryopathy, subchorionic hemorrhage, and fetal bleeding [23]. Given the high thrombotic risk of APS, the fetal toxicity of Coumadin, and the uncertainty of fondaparinux in pregnancy, our patient chose not to become pregnant.

## Figures and Tables

**Procedure 1:**
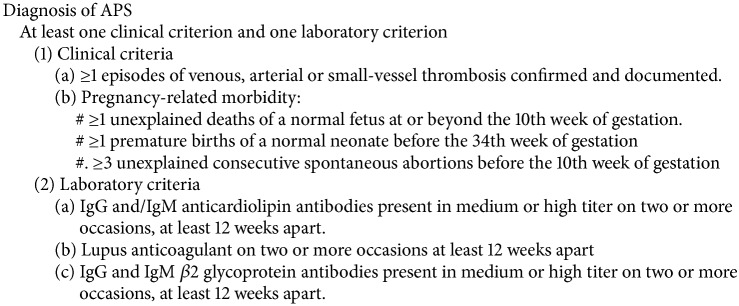
The Sydney classification criteria for antiphospholipid syndrome.

**Table 1:** Antiphospholipid profile at the time of presentation and at twelve-week follow-up.

Tests	At presentation (normal)	Three months after (normal)
Anticardiolipin IgG	140 (<15 GPL units)	135
Anticardiolipin IgM	30.4 (<12.5 MPL units)	60.6
Anti-*β*2 glycoprotein 1 IgG	>150 (<20 SGU)	>150
Anti-*β*2 glycoprotein 1 IgM	38 (<20 SMU)	42
PTT, lupus sensitive	82.5 (32–45 s)	
DRVVT screen	69.1 (31.4–45 s)	
DRVVT confirmation	1.6 (<1.3 s)	
Hexagonal phase phospholipid	39.7 (0–8 s)	
